# Polysaccharide Constituents of Three Types of Sea Urchin Shells and Their Anti-Inflammatory Activities

**DOI:** 10.3390/md13095882

**Published:** 2015-09-16

**Authors:** Heng Jiao, Xiaohui Shang, Qi Dong, Shuang Wang, Xiaoyu Liu, Heng Zheng, Xiaoling Lu

**Affiliations:** 1 Shanghai Medical College, Fudan University, Shanghai 200433, China; E-Mail: 08300240065@fudan.edu.cn; 2 Marine Biopharmaceutical Institute, Second Military Medical University, No. 800, Xiangyin Rd., Shanghai 200433, China; E-Mails: 1141531148@qq.com (X.S.); 769948307@qq.com (Q.D.); 781548697@qq.com (S.W.); biolxy@163.com (X.L.); 3 School of Life Science and Technology, China Pharmaceutical University, No. 24 Tongjiaxiang, Nanjing 210009, China; 4 Department of Biochemistry and Molecular Biology, College of Basic Medical Sciences, Second Military Medical University, Shanghai 200433, China

**Keywords:** sea urchin, polysaccharide, quantitative determination, high performance liquid chromatography, anti-inflammatory

## Abstract

As a source of potent anti-inflammatory traditional medicines, the quantitative chromatographic fingerprints of sea urchin shell polysaccharides were well established via pre-column derivatization high performance liquid chromatography (HPLC) analysis. Based on the quantitative results, the content of fucose and glucose could be used as preliminary distinguishing indicators among three sea urchin shell species. Besides, the anti-inflammatory activities of the polysaccharides from sea urchin shells and their gonads were also determined. The gonad polysaccharide of *Anthocidaris crassispina* showed the most potent anti-inflammatory activity among all samples tested.

## 1. Introduction

Since ancient times in China, dry calcareous shells have been used for Traditional Chinese Medicine. “Chinese Medicinal Herbal” recorded that sea urchins have the function: “Ruan jian san jie, resolving phlegm, eliminating swelling, expectorate sputum accumulation” [1]. According to a modern pharmacological study, it was demonstrated that sea urchin extracts have biological effects, such as anti-cancer, antioxidant, anti-leukemia, anti-fatigue and anti-inflammatory effects [2,3,4]. For example, the compound “Bonellinin” extracted from sea urchin gonad could inhibit the growth of cancer cells [5]. Polyhydroxylated naphthoquinone pigments from the shells of purple sea urchin (*Anthocidaris crassispina*) possessed a strong antiradical activity against 1,1-diphenyl-2-picrylhydrazyl (DPPH), superoxide radical anion, and hydrogen peroxide [6]. For example, polyhydroxylated 1,4-naphthoquinone exhibited dose-dependent antioxidant activities on DPPH radicals [7,8] and inhibited the model of ocular allergic inflammation surpassing the reference drug olopatadine [9]; 6-ethyl-2,3,5,7,8-pentahydroxy-1,4-naphthoquinone (Echinochrome A) existing in sea urchin shells and spines could be used as the auxiliary agent for reducing cardio toxic agent-induced damage [10].

Sea urchin extracts can have many pharmacological effects, but what are the main chemical components? Sea urchins consist of shells and gonads. The sea urchin shells contain minerals (>90%), proteins, polysaccharides and pigments, while sea urchin gonads consist of, for instance, polysaccharides, fatty acid, and proteins [11]. To this end, scholars have paid more attention to the pharmacological activities and chemical constituents of sea urchin gonads. Pozharitskaya *et al.* found that the fractions of ESD1 and ESD2 extracted from gonads of green sea urchin (*Strongylocentrotus droebachiensis*) could be the functional food supplement for the regulation of inflammation and diabetes [12]. It was shown that polysaccharides from the gonad could inhibit tumor transplant and improve immune activity [13]. Ke *et al.* reported *Strongylocentrotus nudus* egg polysaccharides (SEP) displayed antitumor activity mediated by the activation and cytotoxicity of NK cells via TLR2/4 *in vivo* [14]. A water-soluble polysaccharide was extracted from sea urchin intestine, which could significantly inhibit the cell growth [15]. At a dose of 50 μg/mL, the sulfated polysaccharide XB-1 prepared from shells of sea urchin *Hemicentrotus pulcherrimus* could reduce the number of HeLa, MCF-7 and A549 cells to 42.08%, 18.42% and 63.20% [16]. However, there are few reports examining the compositions and inflammatory bioactivities of sea urchin shell polysaccharides.

More than 800 species of sea urchins have been identified in the world, including approximately 100 species in China. In this paper, three common commercial sea urchins, *Strongylocentrotus nudus*, *Anthocidaris crassispina* and *Glyptocidaris crenularis*, were studied from Shandong Peninsula, coastal areas of Zhejiang, Fujian and Guangdong Province, and Liaodong Peninsula, respectively. A quantitative analysis of the composition of shell polysaccharides hydrolysate combined with a chromatographic fingerprint was first established by HPLC-DAD, and the anti-inflammatory activities of polysaccharides of sea urchin shells were reported, which better clarify the structures and anti-inflammatory activities among three species of sea urchin shell.

## 2. Results

### 2.1. Optimization of the Sample Extraction

The polysaccharides of shells were extracted through “the hot water extraction and alcohol precipitation” method, which was optimized by orthogonal design (Table S1). The four parameters (the amount of solvent, the extraction temperature, the extraction time and the number of extraction cycles) were investigated in this experiment (Table S2). As a result, the best method of extraction of sea urchin shell polysaccharides is: a ratio of raw material to water volume of 1:15 (g/mL) (A); an extraction temperature of 70 °C (B); an extraction time of 1 h (C) and undertaking three extraction cycles (D).

### 2.2. Method Validation

**Figure 1:**
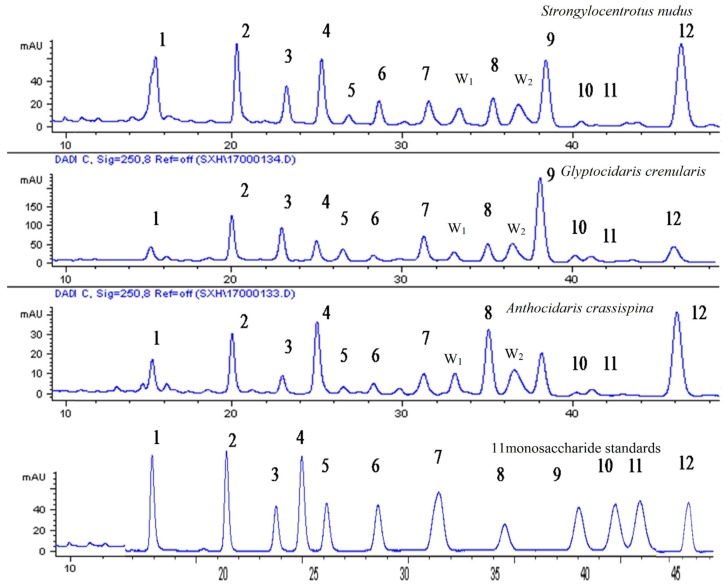
HPLC chromatogram of sea urchin shell polysaccharide hydrolysate. 11 monosaccharide compositions were determined. W_1_ and W_2_ were unknown. 13-Methyl-1-phenyl-2-pyrazolin-5-one (PMP), 2 Mannose (Man), 3 Glucosamine (GlcN), 4 Ribose (Rib), 5 Rhamnose (Rham), 6 Glucuronic acid (GlcUA), 7 Galactosamine (GalN), 8 Glucose (Glc), 9 Galactose (Gal), 10 Xylose (Xyl), 11 Arabinose (Ara), 12 Fucose (Fuc).

The DAD chromatographic profiles of the standard solution and the sample solutions of three species are shown in Figure 1.

#### 2.2.1. Linearity

The standard stock solutions of 11 monosaccharide components were prepared and diluted to six appropriate concentrations and derivatizated with PMP for the HPLC analysis. All calibration curves showed good linearity (*r*^2^ > 0.9990) between the peak area and concentration within test ranges. The regression equation, line range, *r*^2^ and RSD of 11 standard solutions are shown in Table S3.

#### 2.2.2. Precision, Repeatability and Stability

The measurements of 11 monosaccharide compositions were performed with six replicate injections of the same sample. The RSD ranges of peak area of each component are below 3%. Six copies of the samples were prepared separately and analyzed for the repeatability of the HPLC method. The RSD ranges of each sample were 0.75%–2.44%. The peak area of each component at 0, 2, 4, 8, 12 and 24 h was recorded and the RSD at 24 h was lower than 4%, which manifested that the acidolysis product of sea urchin polysaccharides was very stable at room temperature within 24 h.

#### 2.2.3. Recovery

Recoveries were calculated by comparing the determined amount of each component with the amount of originally added. Table S4 showed the method was reproducible with the recovery ranging from 93.75%–107.78% and the RSD below 4.0%.

#### 2.2.4. The Content Determination of 11 Monosaccharide Compositions of 14 Samples

The content of 11 monosaccharide compositions of 14 samples is outlined in Table 1.

### 2.3. Comparison of Different Batches of the Sea Urchin Shells Based on 11 Monosaccharide Components

To distinguish the differences among the monosaccharide composition of polysaccharides of three species of sea urchin shells, Boxplot, PCA and Hierarchical cluster analyses were used.

#### 2.3.1. Boxplot Analysis

**Figure 2:**
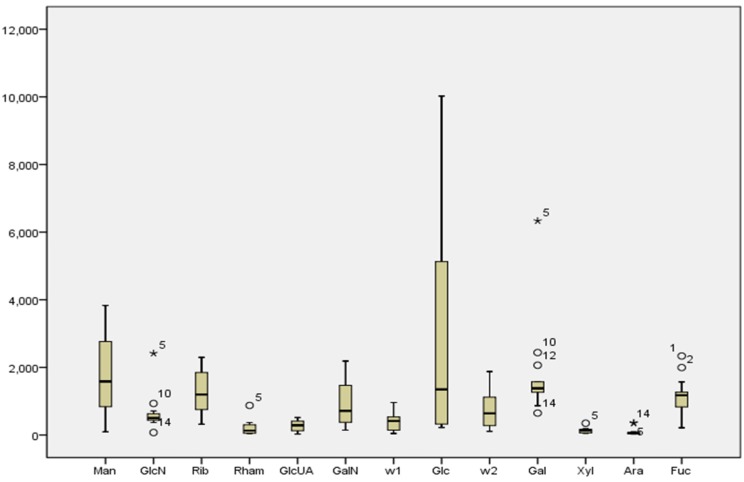
Boxplot of 13 components in different batches of samples. The circle and asterisk were the symbols used for outliers and the number indicates the batch number. Two components (W_1_ and W_2_) were unknown. Sample 1–4 stand for *Strongylocentrotus nudus*. Sample 5, and 7–12 stand for *Glyptocidaris crenularis*. Sample 6, 13 and 14 stand for *Anthocidaris crassispina*.

Boxplot was introduced as a univariate, robust measure to detect the outliers. As shown in Figure 2, the outliers as the individual samples were marked by circles or asterisks. The outliers occur in the sample, which indicate that the samples have inconsistencies with the component content. In other words, the specific points could be potential indexes for the distinction of different species. Six batches of the samples in the content of GlcN, Rham, Gal, Xyl, Ara and Fuc were outliers. The outlier Gal occurs in sample 5, 10, and 12, which all belong to *Glyptocidaris crenularis*; sample 14 from *Anthocidaris crassispina* had the inconsistency in GlcN, Gal and Ara; the content of Fuc in sample 1 and sample 2 from *Strongylocentrotus nudus* were different from one other.

**Table 1:** Contents of 11 components of 14 batches of the sea urchins.

Content (μg/g, Dry Weight)											
No.	Man	GlcN	Rib	Rham	GlcUA	GalN	Glc	Gal	Xyl	Ara	Fuc
*Strongylocentrotus nudus* 1	0.2467	0.1831	0.2111	0.0379	0.1411	0.1221	0.2052	0.2941	0.0333	0.0149	0.6252
*Strongylocentrotus nudus* 2	0.1665	0.1521	0.1640	0.0261	0.1139	0.1305	0.1136	0.2588	0.0562	0.0215	0.5371
*Strongylocentrotus nudus* 3	0.1797	0.1332	0.1336	0.0134	0.1230	0.0971	0.1233	0.1801	0.0122	0.0054	0.2913
*Strongylocentrotus nudus* 4	0.1783	0.1063	0.0977	0.0125	0.0752	0.0946	0.0854	0.1758	0.0079	0.0046	0.3331
*Glyptocidaris crenularis* 5	0.5496	0.7170	0.1996	0.2524	0.0860	0.5836	0.6153	1.2140	0.1344	0.0920	0.4282
*Anthocidaris crassispina*.6	0.5144	0.1550	0.3719	0.1052	0.1326	0.5976	3.9770	0.2759	0.0597	0.0091	0.3331
*Glyptocidaris crenularis* 7	0.6032	0.1277	0.2326	0.0871	0.1028	0.3991	2.3974	0.3099	0.0635	0.0118	0.3258
*Glyptocidaris crenularis* 8	0.8105	0.2074	0.2801	0.0642	0.0375	0.2605	1.8040	0.2859	0.0202	0.0077	0.2000
*Glyptocidaris crenularis* 9	0.5853	0.1440	0.3831	0.0646	0.1117	0.5592	3.6248	0.2626	0.0476	0.0121	0.3180
*Glyptocidaris crenularis* 10	0.7696	0.2739	0.3608	0.0858	0.0336	0.3662	2.0322	0.4742	0.0494	0.0122	0.2377
*Glyptocidaris crenularis* 11	0.2070	0.1050	0.1978	0.0126	0.0484	0.1822	0.6183	0.2709	0.0442	0.0133	0.3280
*Glyptocidaris crenularis* 12	0.3114	0.1475	0.3103	0.0348	0.0617	0.1991	0.4463	0.4032	0.0491	0.0080	0.3502
*Anthocidaris crassispina*.13	0.3627	0.1253	0.0784	0.0137	0.0113	0.0607	0.1426	0.2514	0.0123	0.0050	0.0797
*Anthocidaris crassispina* 14	0.0233	0.0168	0.0634	0.0295	0.0213	0.0325	0.0824	0.1345	0.0105	0.0875	0.2201

#### 2.3.2. PCA Analysis

**Figure 3:**
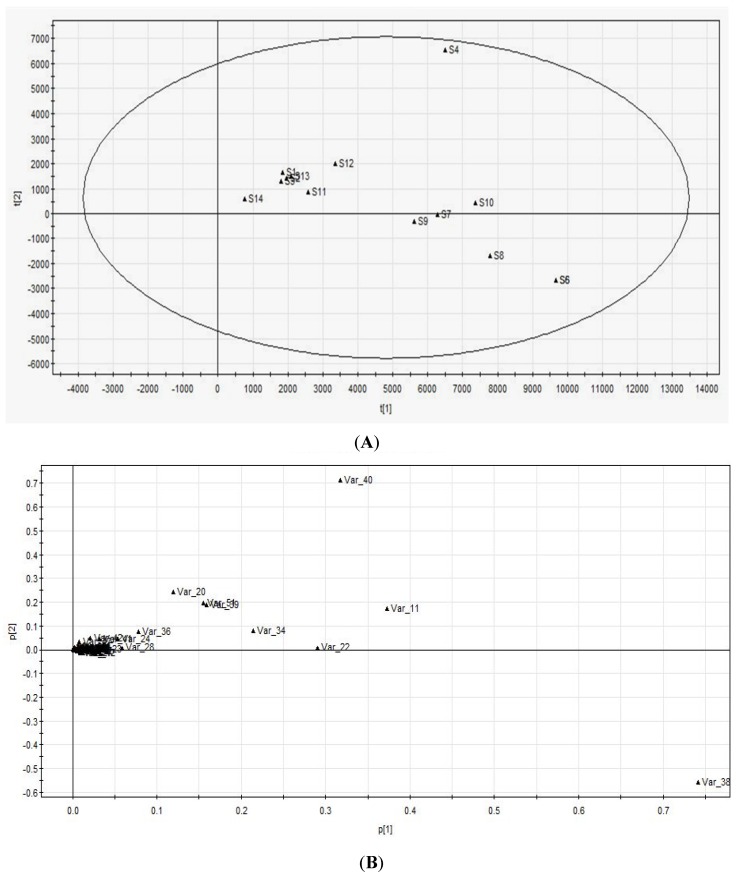
Score (**A**) and loading (**B**) plot of PCA. The numbers 1–14 shown in (**A**) refer to the sample number. The numbers 1–103 shown in (**B**) indicate the chromatographic peaks in HPLC fingerprints. Sample 1–4 stand for *Strongylocentrotus nudus*. Sample 5 and 7–12 stand for *Glyptocidaris crenularis*. Sample 6, 13 and 14 stand for *Anthocidaris crassispina*.

PCA is a very useful method to find which component mainly contributes to the classification among these target samples. It can show us the distribution of the samples in multivariate dimensional space. The PCA score plot was performed by the peak area of every chromatographic peak in the HPLC fingerprint. The weighted value of the variable farther from the origin point is larger, which could make a larger contribution to the classification of the samples. In Figure 3A, the samples from the same district gathered in the same region. For example, the bath numbers 1–3, 11 and 12 of sea urchin shells from Shandong Province (except 4) gathered together. Samples belonging to the same genus distributed in the same region as well. For example, samples 7–10 distributed in the same region all belong to *Glyptocidaris crenularis*. As shown in Figure 3B, Var_38 (Glc), Var_11 (Man), Var_40 (Gal), Var_22 (Rib), Var_34 (GalN) and Var_51 (Fuc) might be the primary components.

#### 2.3.3. Hierarchical Cluster Analysis

**Figure 4:**
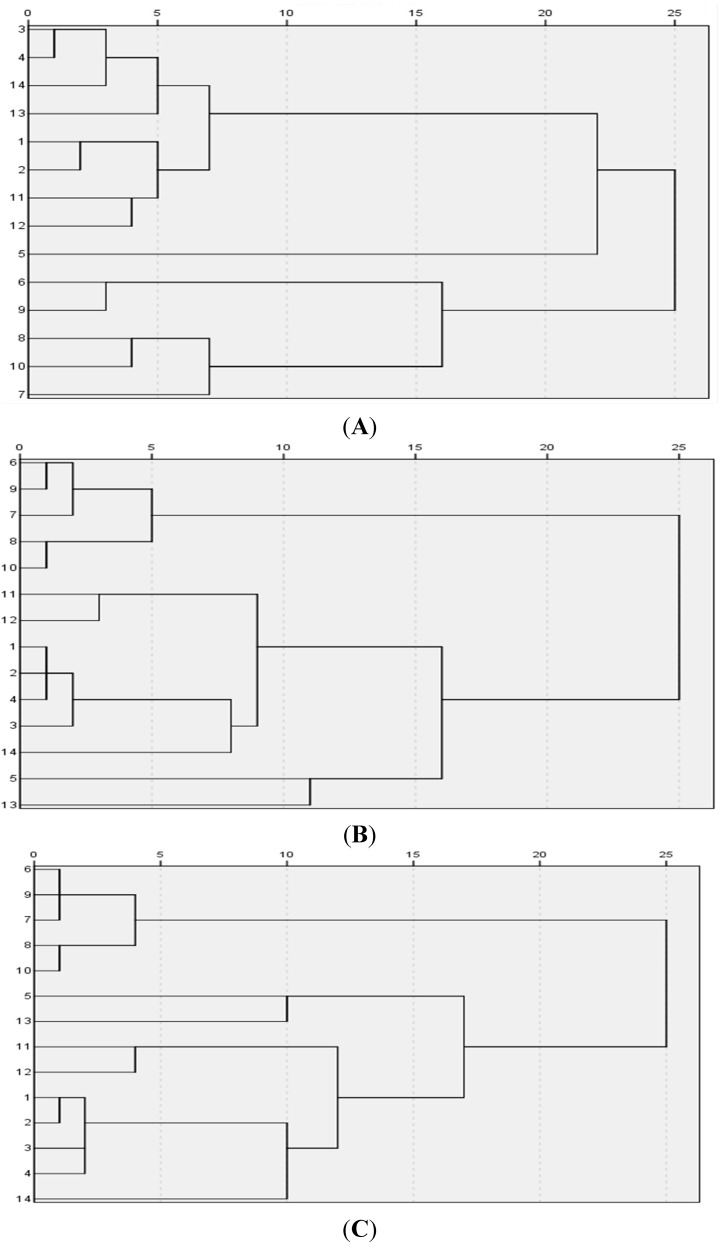
Hierarchical cluster analysis based on between-groups the average-linkage method. Dendrograms in (**A**–**C**) were based on the areas of common peaks in fingerprints with three similarity algorithms: Euclidean distance (**A**); cosine of angle (**B**) and Pearson correlation (**C**). Sample 1–4 stand for *Strongylocentrotus nudus*. Sample 5 and 7–12 stand for *Glyptocidaris crenularis*. Sample 6, 13 and 14 stand for *Anthocidaris crassispina*.

Hierarchical cluster analysis was introduced to make a further illustration of the comparison of the samples. The analysis was performed among groups by the average-linkage method. The Euclidean distance (A); cosine of angle (B) and Pearson correlation (C) were used to measure the closeness of the contents of eleven components among different batches. Compared with three dendrograms shown in Figure 4, the cluster based on the cosine of angle (B) and Pearson correlation (C) were similar and conformed to the authentic classifications: samples 1–4 belonged to *Strongylocentrotus nudus*; samples 7–10 belonged to *Glyptocidaris crenularis*; samples 11 and 12 belonged to *Glyptocidaris crenularis*. The results of the cosine of angle (B) and Pearson correlation (C) analyses were different from the Euclidean distance (A) analysis because of their own different features. The Pearson correlation paid attention to every peak area, in other words, each peak made an equal contribution to the final similarity. However, the cosine of angle (B) and Pearson correlation (C) were sensitive to the large peak area. Sample 5 was not grouped with samples 7–10. Samples 6, 13 and 14 were not in the same class; moreover, the distance of sample 6 was far from samples 13 and 14 in the dendrogram because the statistical results were affected by the collection area and sample size.

### 2.4. Similarity Analysis of HPLC Fingerprints of 14 Batches of Sea Urchin Shells

Similarity analysis was performed by Chromatographic Fingerprint of Traditional Chinese Medicine. As shown in Table S5, the value of similarity of every sample was above 0.8, which demonstrated that most samples have high similarity, such as samples 1–4. The similarity of sample 9 and sample 14 was under 0.8, which might be caused by collection intensity.

### 2.5. The Content Analysis of 11 Monosaccharide Compositions of 14 Samples

As shown in Table 1, the content range interval of 11 monosaccharide compositions was large, from 1.0795–3.8946. The content of Glc changed a lot with species diversity: *Glyptocidaris crenularis* > *Anthocidaris crassispina* > *Strongylocentrotus nudus*, and the content of Glc in *Glyptocidaris crenularis* was nearly five times that in *Strongylocentrotus nudus*. Moreover, the average content of Xyl was less than 2% and next to Ara. As shown in Table 1, the content of each monosaccharide in each sample from the same species of sea urchin was similar, but the average content of each monosaccharide in different genus was notably different. In other words, monosaccharide content was greatly influenced by the genus of the sea urchin.

### 2.6. Comparisons between the Hydrolysate Compositions of the Sea Urchin Shell and Gonad Polysaccharide

**Figure 5:**
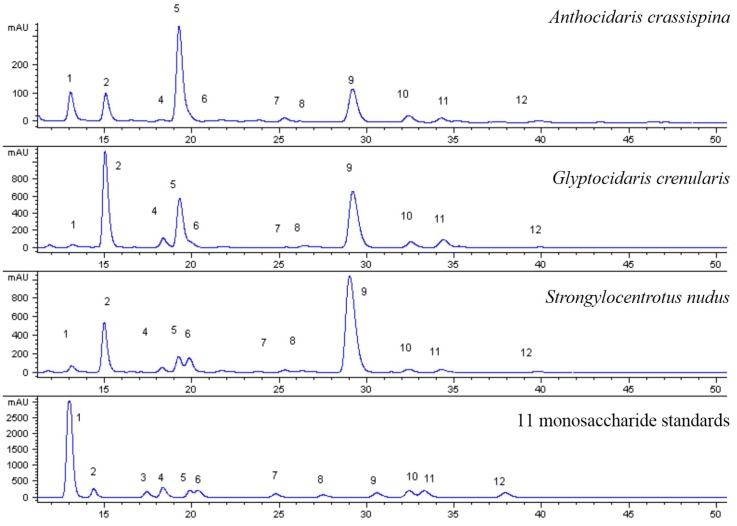
HPLC chromatograms of the sea urchin gonad polysaccharide. 1 PMP, 2 Man, 3 GlcN, 4 Rib, 5 Rham, 6 GlcUA, 7 GalN, 8 Glc, 9 Gal, 10 Xyl, 11 Ara, 12 Fuc.

In Figure 1 and Figure 5, compositions of sea urchin shell polysaccharide were similar to gonad polysaccharides. They contained common monosaccharide fragments, such as Man, Rib, Rham, GalN, Glc, Gal, Ara and Fuc. These common fragments decided the structural similarity between shell polysaccharide and gonad polysaccharide. Moreover, shell polysaccharide contained glucosamine and two unknown components.

### 2.7. Effect of the Sea Urchin Polysaccharide (Shells and Gonads) on Cell Viability

**Figure 6:**
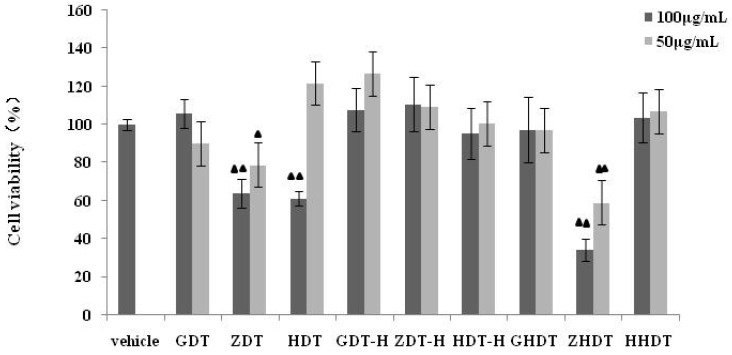
Effect of the sea urchin shell and gonad polysaccharides on RAW264.7 cell viability. Compared with the Vehicle group ^▲▲^ *p* < 0.01. GDT*-Strongylocentrotus nudus* shell polysaccharide, GDT-H-*Strongylocentrotus nudus* shell polysaccharide hydrolysate, ZDT*-Anthocidaris crassispina* shell polysaccharide, ZDT*-*H-*Anthocidaris crassispina* shell polysaccharide hydrolysate, HDT*-Glyptocidaris crenularis* shell polysaccharide, HDT-H*-Glyptocidaris crenularis* shell polysaccharide hydrolysate.

As shown in Figure 6, there were some significant differences in the growth inhibition of the murine macrophage cell line RAW264.7 among three species of the sea urchin (*p* < 0.01).The polysaccharides of the GDT and HDT groups (50 μg/mL) had no obvious toxicity. However, HDT (100 μg/mL) and ZDT showed an inhibition of nearly 30%. As expected, the hydrolysate of shell polysaccharides (GDT-H group, HDT-H group and ZDT-H group) had no cytotoxic effect on the cell growth. As for the sea urchin gonads, the polysaccharide of *Anthocidaris crassispina* gonad exhibited a larger inhibition effect on the cells than did two species of sea urchin gonads.

### 2.8. Effect of the Sea Urchin Shell Polysaccharide on LPS-Stimulated Cell Viability

**Figure 7:**
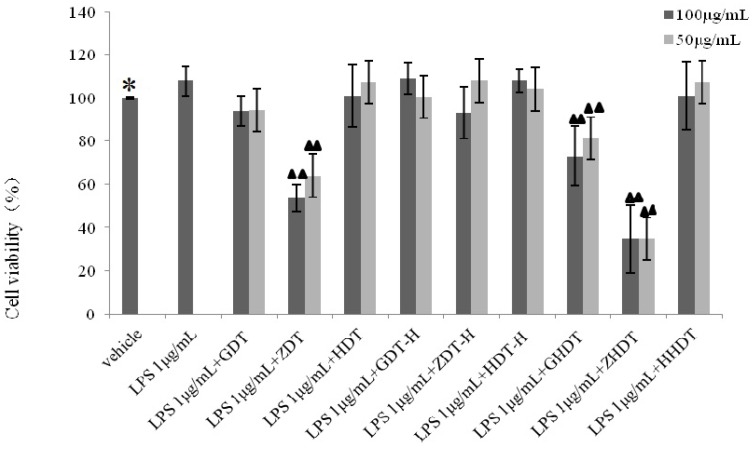
Effect of the sea urchin shell and gonad polysaccharides on LPS-stimulated RAW264.7 cell viability. Compared with the vehicle, * *p* < 0.01; compared with the LPS group, ^▲▲^ *p*<0.01. GDT*-Strongylocentrotus nudus* shell polysaccharide, GDT-H-*Strongylocentrotus nudus* shell polysaccharide hydrolysate, ZDT*-Anthocidaris crassispina* shell polysaccharide, ZDT*-*H-*Anthocidaris crassispina* shell polysaccharide hydrolysate, HDT*-Glyptocidaris crenularis* shell polysaccharide, HDT-H*-Glyptocidaris crenularis* shell polysaccharide hydrolysate.

As shown in Figure 7, the cells were incubated for 48 h in LPS solution without the samples, the vitality of which increased to 107.76% with the comparison to the control group (*p* < 0.01). Compared with the LPS group, GDT and HDT had little inhibition effect. ZDT (100 μg/mL, 50 μg/mL) could significantly prevent LPS-stimulated cell proliferation (*p* < 0.01). In addition, there was an inhibition of 70% in the ZHDT group. The cell viability of GHDT group reduced nearly 30%. However, the hydrolysate (GDT-H, HDT-H and ZDT-H) of the shell polysaccharide showed no cytotoxicity to Raw264.7 cells.

**Figure 8:**
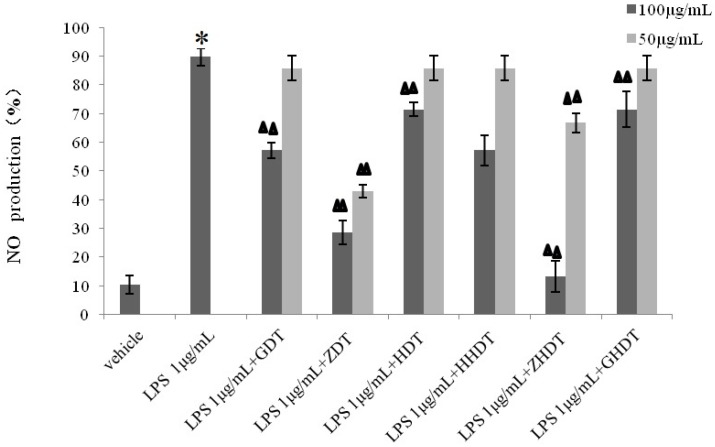
Effect of the sea urchin shell and gonad polysaccharides on LPS-stimulated NO production. Compared with the vehicle group, * *p* < 0.01; compared with the LPS group, ^▲▲^ *p* < 0.01. GDT*-Strongylocentrotus nudus* shell polysaccharide, GDT-H-*Strongylocentrotus nudus* shell polysaccharide hydrolysate, ZDT*-Anthocidaris crassispina* shell polysaccharide, ZDT*-*H-*Anthocidaris crassispina* shell polysaccharide hydrolysate, HDT*-Glyptocidaris crenularis* shell polysaccharide, HDT-H*-Glyptocidaris crenularis* shell polysaccharide hydrolysate.

### 2.9. Effect of the Sea Urchin Shell Polysaccharide on NO Production in RAW264.7 Cells

Compared with the vehicle group, NO production released by RAW264.7 increased to 89.67% ± 2.72%, which indicated that the inflammatory model was successfully built. Incubating the samples with LPS for 36 h, GDT and HDT (100 μg/mL) showed slightly inhibitory effect on LPS-induced nitrite secretion. In Figure 8, LPS-induced NO production reduced to 40% at concentration of 100, 50 μg/mL ZDT. Moreover, the gonad polysaccharide of three species of the sea urchin shells at the high concentration (GHDT, HHDT and ZHDT) showed strong inhibition to LPS-stimulated NO production (*p* < 0.01).Furthermore, ZHDT could also repress the release of nitrite secretion at 50 μg/mL.

## 3. Discussion

According to the record of an ancient book, sea urchin shells can diminish inflammation and relieve pain. However, there has been no research investigating the anti-inflammatory activity of sea urchin shells. In this paper, an inflammation model of murine macrophage cell line RAW264.7 was first established to explore the activity of three species of sea urchin shell polysaccharides. The experiment demonstrated that the polysaccharide of the purple sea urchin shell and gonad showed an obvious inhibitory effect on RAW264.7 cell growth. Polysaccharides from the purple sea urchin shell and gonad and *Strongylocentrotus nudus* gonad had different inhibition actions on the proliferation of RAW264.7 cells stimulated by LPS. The inhibitory activity of purple sea urchin polysaccharides exceeded those of the other sea urchin, and the polysaccharide of *Glyptocidaris crenularis* has no anti-inflammatory effect. In experiments measuring the release of NO, we found that every genus of sea urchin shell polysaccharide showed different inhibitory functions: the inhibition rate of shell polysaccharides of *Anthocidaris Crassispina* (70%) was greater than the inhibition rate of *Strongylocentrotus nudus* shell polysaccharide (40%), which in turn was greater than the inhibition rate of shell polysaccharide of *Glyptocidaris crenularis* (20%). Next, we analyzed the monosaccharide compositions of the polysaccharides of three types of sea urchin shells. As shown in Table 1, the monosaccharide compositions were similar, but the contents of each monosaccharide were different. For example, the amount of fucose and glucuronic acid in *Strongylocentrotus nudus* shells exceeded 25% and 5%, respectively. In addition, compared with other sea urchin shells, the amount of glucose of *Glyptocidaris crenularis* was larger than others, which reached more than 30%. Thus, we could deduce that differences of the primary monosaccharide composition led to the varying anti-inflammatory activity of the polysaccharide of sea urchin shell.

Based on the above experimental results, we summarized the difference of anti-inflammatory activity between the purple sea urchin shell and gonad polysaccharide. As shown in Figure 6, Figure 7 and Figure 8, polysaccharides from the purple sea urchin shell (100 μg/mL) and gonads (100 μg/mL) produced inhibition rates of 40% and 60% on RAW264.7 cells without LPS stimulation. Meanwhile, the purple sea urchin shell polysaccharide and gonad polysaccharide reduced the amount of RAW264.7 cells stimulated by LPS to 50% and 60%, and effectively inhibited the release of NO with inhibition rates of 70% and 80%. However, the polysaccharide hydrolysate of the purple sea urchin shell exhibited no inhibition to RAW264.7 cell proliferation.

It was determined that compositions of purple sea urchin shell polysaccharide were similar to its gonad polysaccharides, and both of them were neutral heteropolysaccharides. In Figure 1 and Figure 5, they contained common monosaccharide fragments, such as Man, Rib, Rham, GalN, Glc, Gal, Ara and Fuc. These common fragments decided the structural similarity between shell polysaccharide and gonad polysaccharide. Shell polysaccharide contained glucosamine and two unknown components. In general, glucosamine plays a role in boosting the growth of bone cells, and therefore mainly exists in the animal shell. Two unknown components need to be further confirmed. According to the literature, CR3, the human macrophage surface polysaccharide receptor, has specificity to mannose, dextran and *N*-acetyl-glucosamine. This specific binding can promote the phagocytosis by macrophages and inhibit the generation of the NOS enzyme [17,18]. As is widely known, both sea urchin shell and gonad polysaccharides contained glucose and mannose. Therefore, the commonalities of the structure and content of the monosaccharide compositions determine the anti-inflammatory activity, but the differences of their contents explain the differences in anti-inflammatory activity. In addition, the connection of the glycosidic bond and spatial structure between monosaccharide and monosaccharide affected the activities of various species of sea urchin polysaccharides, and the anti-inflammatory activities of the polysaccharide of the purple sea urchin gonad and shell. In experiments of anti-inflammatory activity, we found the polysaccharide of purple sea urchin shell and gonads were cytotoxic to RAW264.7 cells. Determining whether the anti-inflammatory activities of the polysaccharide of the purple sea urchin were produced directly by itself or indirectly by inhibiting cell growth relies on further study of the anti-inflammatory mechanism.

There are many types of sea urchins in the world, but how to distinguish them? This paper established the characteristic fingerprints of three species of sea urchin shell polysaccharide hydrolysate and determined the contents of 11 monosaccharide compositions in 14 batches of sea urchin shells. Then, it was shown that three species of sea urchin shells were consistent with the monosaccharide compositions, but exhibited differences in content. For example, the content of Fuc, Gal and Man of *Strongylocentrotus nudus* is higher than other compositions; purple sea urchin mainly consisted of Glc, Man and Fuc; the primary monosaccharide compositions of *Glyptocidaris crenularis* were Glc, Man and Gal. In *Strongylocentrotus nudus*, the content of fucose exceeded 20%, however, it was below 15% in *Anthocidaris crassispina* and *Glyptocidaris crenularis*. Thus, fucose can be used as an indicator for distinguishing among species of sea urchin. Furthermore, there was a gap in the content of glucose between *Anthocidaris crassispina* and *Glyptocidaris crenularis*, its content was mostly over 20% in purple sea urchin and below 20% in *Glyptocidaris crenularis*, so glucose was another index for the sea urchin. Besides, diversities of the structures of polysaccharides were also affected by specificity of sea urchin shells, environmental conditions and very low content of polysaccharides in samples.

## 4. Experimental Section

### 4.1. Samples

Fourteen batches of sea urchins were harvested from the north to the south (Table 2). These samples were identified by Professor Xiaoqi Zeng, Ocean University of China. The collected sea urchins were eviscerated, washed and then air dried. Dried shells were packed and frozen at −20 °C. The voucher specimens (No. 130806, No. 121024, No. 131016) were deposited at the department of Biochemistry and Molecular Biology, Second Military Medical University, Shanghai, China.

**Table 2:** Summary of the tested samples.

Sample Number	Species (Family)	Popular Names	Province	Harvesting Time
1	*Strongylocentrotus nudus*	Dalian purple sea urchin or Blackthorn pot	Shandong	6 August 2013
2	*Strongylocentrotus nudus*		Shandong	6 August 2013
3	*Strongylocentrotus nudus*		Shandong	6. August 2013
4	*Strongylocentrotus nudus*		Shandong	6. August 2013
5	*Glyptocidaris crenularis*	/	Shandong	25 March 2013
6	*Anthocidaris crassispina*	Sea needle or Sea chestnuts	Zhejiang	24 September 2012
7	*Glyptocidaris crenularis*	/	Dalian	24 October 2012
8	*Glyptocidaris crenularis*	/	Dalian	8 November 2012
9	*Glyptocidaris crenularis*	/	Dalian	23 November 2012
10	*Glyptocidaris crenularis*	/	Dalian	10 December 2012
11	*Glyptocidaris crenularis*	/	Shandong	8 August 2013
12	*Glyptocidaris crenularis*	/	Shandong	8 August 2013
13	*Anthocidaris crassispina*	Sea needle or Sea chestnuts	Guangdong	15 August 2013
14	*Anthocidaris crassispina*	Sea needle or Sea chestnuts	Guangdong	16 October 2013

### 4.2. Chemicals

Acetonitrile (HPLC grade), 3-Methyl-1-phenyl-2-pyrazolin-5-one (PMP, AR), trifluoroacetic acid (TFA, AR), trichloromethane (AR), HCl (AR), and NaOH (AR) were purchased from Sinopharm Chemical Reagent Co., Ltd. (Shanghai, China). Deionized water was obtained from a Hi-tech K-flow water purification system (Shanghai, China). Standard substances including Mannose (d-Man), Glucose (d-Glc), Galactose (d-Gal), Rhamnose (l-Rham), Xylose (d-Xly), Arabinose (l-Ara) were purchased from Sinopharm Chemical Reagent Co., Ltd. (Shanghai, China). Ribose (d-Rib), Fucose (l-Fuc) and Glucuronic acid (d-GlcUA) were purchased from Yuanye Biology Co., Ltd. (Shanghai, China). The purities of all the standards were above 98% as determined by HPLC.

LPS (*Escherichia coli* 055:B5) was purchased from Sigma. Fetal bovine serum (FBS), Trypsin-EDTA and DMEM with antibiotics (100 U/mL penicillin, 100 μg/mL streptomycin) were purchased from Beijing Solarbia Science and Technology Co., Ltd. (Beijing, China). A CCK-8 (Cell Counting Kit-8) was obtained from Obio Technology Co., Ltd. (Shanghai, China). A Nitric Oxide (NO) assay kit (Chemical NO-assay) was purchased from Nanjing Jiancheng Bioengineering Institute.

### 4.3. Preparation of Saccharide Derivatization Reagents

Dried sea urchin shells were crushed into powder. Fifty grams of powder was weighed and soaked in 750 mL water, extracted three times with a heating circumfluence method at 70 °C for 1 h each time, and filtered. The filtrate was combined and evaporated to 50 mL in a DK-8 Delectric water bath (Shanghai Jinghong Laboratory Instrument Co., Ltd., Shanghai, China), then 250 mL absolute ethyl alcohol was added for precipitation at room temperature and centrifuged at 5000 rpm for 10 min. The precipitate was dried and stored at 4 °C. Fifty milligrams of powder was accurately weighed and poured into the glass test tube, then added to 1 mL TFA. The mixture was boiled for 6 h with an oil-bath method and then evaporated to dryness by a rotary evaporator (Shanghai Ailang Instruments Co., Ltd., Shanghai, China).The acidolysis product was dissolved with 2 mL water and filtered through a 0.45 μm microporous membrane to remove impurities. Then, 100 μL polysaccharide hydrolysate, 50 μL NaOH (0.2 mol/L) and 40 μL PMP were reacted for 30 min by water bath at 70 °C. After returning to room temperature and neutralization for hydrochloric acid, the redundant derivatization reagent was extracted with the addition of 200 μL chloroform and centrifuged at 8000 rpm for 20 min, filtered through a 0.45 μm microporous membrane to yield the sample solution. Each sample was determined in triplicate.

### 4.4. Preparation of Standard Solutions

Eleven monosaccharide (Mannose, Glucose, Galactose, Rhamnose, Xylose, Arabinose, Ribose, Fucose, Glucuronic acid, Glucosamine and Galactosamine) standards were accurately weighed and dissolved in volumetric flasks to obtain appropriate stock solutions, respectively. All standard solutions were prepared in water and stored at 4 °C before use.

### 4.5. Analytical Method

A chromatographic separation was performed on an Agilent 1100 series HPLC-DAD system equipped with a vacuum degasser, a binary pump, a column compartment and a diode array detector (DAD). All compositions of the samples were injected on a CNW™ C_18_ column (250 mm × 4.6 mm, 5 μm) at 30 °C. The mobile phase was composed of solvent A (acetonitrile) and solvent B (PBS, pH 6.8) with a linear gradient: 0–25 min (A 16%–18%), 25–45 min (A 18%–19%), and 45–50 min (A 19%–19%) with a flow rate of 1 mL/min. The detection wavelength was set at 250 nm and the sample injection volume was 30 μL.

### 4.6. Method Validation

The performance of the quantitative method used to determine 11 monosaccharide components in sea urchin shells was established through a validation protocol that followed the appendix of China Pharmacopeia (2010 edition, Volume I). The parameters of linearity, precision, repeatability stability, and recovery accuracy were evaluated.

#### 4.6.1. Linearity

These stock solutions were diluted to a series of mixed working solutions with different concentrations. Calibration curves were established based on six levels for each in triplicate. The curves’ regression equations were calculated in the form of *y* = a*x* + b. *y* represented the peak area and *x* represented the compound concentration.

#### 4.6.2. Precision, Repeatability and Stability

The precision was examined by performing assays with six replicate injections of sample 1. Six replicate sample solutions were independently prepared and analyzed to confirm the repeatability of the method. The stability was assessed by analyzing the same sample solution at 0, 2, 4, 8, 12 and 24 h.

#### 4.6.3. Recovery

The recovery test was investigated with a standard addition method. Three different concentration levels (approximately equivalent to 0.8, 1.0 and 1.2 times the concentration of sample 1) of the mixed standard solution were added into the sample 1 solution.

### 4.7. Quantitative Determination of 11 Monosaccharide Components in Sea Urchin Shells

Eleven known compositions were determined in 14 batches of the sea urchin shells. The peak area of each component was recorded and the corresponding concentration calculated according to the calibration curve. Each sample was evaluated in triplicate. The content percentages of each component of three species of the sea urchin shell polysaccharide were written in Table 1.

### 4.8. Establishment of the HPLC Fingerprints of 14 Batches of the Sea Urchin Shells

The HPLC fingerprints of 14 batches of samples after peak alignment are established by Chromatographic Fingerprint of Traditional Chinese Medicine. The reference fingerprint of different batches of samples was generated by similarity analysis software for Chromatographic Fingerprint of Traditional Chinese Medicine, which was recommended by State Food and Drug Administration of China (SFDA).

### 4.9. Anti-Inflammatory Activity

#### 4.9.1. Cell Culture

The murine macrophage cell line RAW264.7 was cultured in the flask in Dulbecco’s modified Eagle’s medium (DMEM) with 10% fetal bovine serum (FBS). The cells were incubated at 37 °C under 5% CO_2_ humidified air.

#### 4.9.2. Cell Viability Assay

The cells were seeded in 96-well plates for 24 h and the old medium changed with various concentrations of the sea urchin shell and gonads polysaccharides (100 μg/mL, 50 μg/mL) for 48 h at 37 °C. Using the cell counting kit-8 (CCK-8), CCK-8 solution was added to 96 wells and the cells were incubated for half an hour. The optical density was read at 450 nm with an ELISA reader (550 ELISA Reader, Bio-Rad Co., Ltd., Hercules, CA, USA).

#### 4.9.3. Cell Viability Assay after LPS-Induced Raw264.7

The cells were plated in 96-well plates for 24 h. The medium was discarded and LPS (1 μg/mL) was added to every well except for the vehicle group. Next, the cells were incubated for 48 h at 37 °C. The optical density was read at 450 nm with an ELISA reader.

#### 4.9.4. Determination of NO Production

The cells were plated in 24-well plates for 24 h. The medium was discarded. Cells were incubated with LPS (1 μg/mL) and the varying concentrations of the sea urchin shell polysaccharide (100 μg/mL, 50 μg/mL) solutions for 36 h. The supernatant was treated as the Nitric Oxide (NO) assay kit. Then the absorbance was measured at 570 nm with an ELISA reader. The assays were performed in triple and the mean and standard deviation (SD) were calculated. The amount of NO production was calculated by the following formula: NO (μmol/L) = (*A*_i_ − *A*_o_)/(*A*_s_ − *A*_o_) × 20 (μmol/L) × *d*, where A_o_ is the absorbance with the vehicle group, *A*_i_ is absorbance with the sample group, A_s_ is the absorbance with the NO standard solution (20 μmol/L), and *d* is the diluted multiples. The percentage of NO production was calculated by the following formula: NO% = (*A*_i_ − *A*_o_)/(*A*_L_ − *A*_o_) × 100%, where *A*_o_ is the absorbance with the vehicle group, and A_i_ is absorbance with the sample group, A_L_ is the absorbance with the LPS group.

### 4.10. Data Statistics

The relative standard deviations (RSD) of eleven components were calculated in Excel. Boxplot and Hierarchical cluster analyses were performed by IBM SPSS Statistics. PCA was performed by SIMCA-P12.0.

## 5. Conclusions

To the best of our knowledge, this report is the first to establish a RAW264.7 anti-inflammatory model for sea urchin shell polysaccharide. The results of anti-inflammatory activity showed that purple sea urchin polysaccharide generated obvious inhibitory effects on the growth of RAW264.7 cells and the release of NO. We concluded that the anti-inflammatory activity of the polysaccharide was primarily related with the connecting style and space structure of the glycosidic bond, particularly with glucose and mannose. Through analysis of content determination of principal components, the author concluded that the difference in content of fucose and glucose could be used for preliminarily distinguishing three species of sea urchin shell, which laid the groundwork for the establishment of the sea urchin shell quality standard. Clearly, due to the collection region and the limitation of sample sizes, establishing quality standards of all types of sea urchins warrant further study and validation.

## Supplementary Files

Supplementary File 1
