# Supplementary material for: Polysaccharide Constituents of Three Types of Sea Urchin Shells and Their Anti-Inflammatory Activities
Source: Mar Drugs. 2015 Sep 16;13(9):5882–900. doi: 10.3390/md13095882 (PMC4584359; doi:10.3390/md13095882)
Supplement: Supplementary File 1 [file marinedrugs-13-05882-s001.docx]

**Supplementary Information**

**Table S1.** Experimental factors and levels.

| **Factors** | | | | |
| --- | --- | --- | --- | --- |
| **Level** | **A** | **B(°C)** | **C (h)** | **D** |
| 1 | 1:5 | 40 | 1 | 1 |
| 2 | 1:10 | 70 | 2 | 2 |
| 3 | 1:15 | 90 | 3 | 3 |

**Table S2.** Results of the orthogonal experiment.

| **No.** | **Factors** | | | | **Yield of Polysaccharide (%)** |
| --- | --- | --- | --- | --- | --- |
|  | **A** | **B** | **C** | **D** |  |
| 1 | 1 | 1 | 1 | 1 | 0.104 |
| 2 | 1 | 2 | 2 | 2 | 0.275 |
| 3 | 1 | 3 | 3 | 3 | 0.189 |
| 4 | 2 | 1 | 2 | 3 | 0.264 |
| 5 | 2 | 2 | 3 | 1 | 0.159 |
| 6 | 2 | 3 | 1 | 2 | 0.210 |
| 7 | 3 | 1 | 3 | 2 | 0.162 |
| 8 | 3 | 2 | 1 | 3 | 0.292 |
| 9 | 3 | 3 | 2 | 1 | 0.197 |
| *K*_1_ | 0.568 | 0.530 | 0.606 | 0.460 |  |
| *K*_2_ | 0.633 | 0.726 | 0.736 | 0.647 |  |
| *K*_3_ | 0.651 | 0.596 | 0.510 | 0.745 |  |
| $\overline{K_{1}}$ | 0.189 | 0.177 | 0.245 | 0.153 |  |
| $\overline{K_{2}}$ | 0.211 | 0.242 | 0.202 | 0.216 |  |
| $\overline{K_{3}}$ | 0.217 | 0.199 | 0.170 | 0.248 |  |
| *R* | 0.028 | 0.065 | 0.075 | 0.095 |  |

**Table S3.** Calibration curves, precision, repeatability and stability of 11 types
of monosaccharides.

| **Analyses** | **Calibration Curves (*n*** **=** **6)** | **Linearity Ranges (mg/mL)** | **R (*n*** **=** **6)** | **Precision (RSD%)** | **Repeatability (RSD%)** | **Stability** |
| --- | --- | --- | --- | --- | --- | --- |
| Man | y = 47482x − 15.27 | 0.001563~0.1000 | 0.9989 | 1.70 | 1.82 | 2.77 |
| GlcN | y = 33435x + 17.40 | 0.001953~0.3125 | 0.9999 | 0.89 | 3.37 | 2.87 |
| Rib | y = 61822x − 71.15 | 0.001563~0.1000 | 0.9989 | 1.74 | 2.24 | 1.85 |
| Rham | y = 34366x + 6.15 | 0.0007813~0.05000 | 0.9997 | 2.83 | 0.23 | 1.65 |
| GlcUA | y = 37555x − 14.48 | 0.0007813~0.05000 | 0.9998 | 1.97 | 1.14 | 1.39 |
| GalN | y = 36155x + 25.53 | 0.001563~0.1000 | 0.9996 | 1.00 | 1.58 | 3.12 |
| Glc | y = 25103x + 14.00 | 0.007813~0.5000 | 0.9998 | 1.79 | 3.46 | 3.27 |
| Gal | y = 52656x − 59.63 | 0.006250~0.2000 | 0.9989 | 1.75 | 1.75 | 3.59 |
| Xyl | y = 23324x + 34.81 | 0.0007813~0.05000 | 0.9989 | 2.11 | 2.06 | 1.39 |
| Ara | y = 38214x + 11.58 | 0.0007813~0.05000 | 0.9998 | 3.47 | 2.47 | 2.89 |
| Fuc | *y* = 38906x − 95.39 | 0.001563~0.1000 | 0.9989 | 3.18 | 2.55 | 3.41 |

**Table S4.** Recoveries of the method.

| **Analyses** | **Initial (mg/mL)** | **Added (mg/mL)** | **Detected (mg/mL)** | **Recovery (%)** | **RSD (%)** |
| --- | --- | --- | --- | --- | --- |
| Man | 0.0510 | 0.0408 | 0.0909 | 97.79 | 1.96 |
|  |  | 0.0510 | 0.0990 | 94.12 |  |
|  |  | 0.0612 | 0.1102 | 96.73 |  |
| GlcN | 0.0770 | 0.0616 | 0.1392 | 100.97 | 0.05 |
|  |  | 0.0770 | 0.1547 | 100.91 |  |
|  |  | 0.0924 | 0.1702 | 100.87 |  |
| Rib | 0.0200 | 0.0160 | 0.0350 | 93.75 | 0.05 |
|  |  | 0.0200 | 0.0388 | 94.00 |  |
|  |  | 0.0240 | 0.0425 | 93.75 |  |
| Rham | 0.0250 | 0.0200 | 0.0456 | 103.00 | 1.54 |
|  |  | 0.0250 | 0.0507 | 102.80 |  |
|  |  | 0.0300 | 0.0567 | 105.67 |  |
| GlcUA | 0.0090 | 0.0072 | 0.0158 | 94.44 | 2.32 |
|  |  | 0.0090 | 0.0179 | 98.89 |  |
|  |  | 0.0106 | 0.0192 | 96.23 |  |
| Glc | 0.0620 | 0.0500 | 0.1115 | 99.00 | 0.77 |
|  |  | 0.0620 | 0.1239 | 99.84 |  |
|  |  | 0.0740 | 0.1364 | 100.54 |  |
| Gal | 0.1210 | 0.0970 | 0.2176 | 99.59 | 0.11 |
|  |  | 0.1210 | 0.2417 | 99.75 |  |
|  |  | 0.1450 | 0.2657 | 99.79 |  |
| Xyl | 0.0130 | 0.0108 | 0.0244 | 105.56 | 1.55 |
|  |  | 0.0130 | 0.0264 | 103.08 |  |
|  |  | 0.0156 | 0.0290 | 102.56 |  |
| Ara | 0.0090 | 0.0074 | 0.0168 | 105.41 | 1.28 |
|  |  | 0.0090 | 0.0187 | 107.78 |  |
|  |  | 0.0110 | 0.0206 | 105.45 |  |
| Fuc | 0.0430 | 0.0340 | 0.0751 | 94.41 | 0.72 |
|  |  | 0.0430 | 0.0832 | 93.49 |  |
|  |  | 0.0520 | 0.0923 | 94.81 |  |

**Table S5.** Similarity analysis of 14 batches of sea urchin shells.

| **NO.** | **Similarity (S)** | **NO.** | **Similarity (S)** |
| --- | --- | --- | --- |
| 1 | 0.913 | 8 | 0.869 |
| 2 | 0.883 | 9 | 0.773 |
| 3 | 0.926 | 10 | 0.894 |
| 4 | 0.916 | 11 | 0.956 |
| 5 | 0.911 | 12 | 0.966 |
| 6 | 0.890 | 13 | 0.866 |
| 7 | 0.842 | 14 | 0.743 |

© 2015 by the authors; licensee MDPI, Basel, Switzerland. This article is an open access article distributed under the terms and conditions of the Creative Commons Attribution license (http://creativecommons.org/licenses/by/4.0/).
